# Estimating the Health and Economic Benefits of Universal Salt Iodization Programs to Correct Iodine Deficiency Disorders

**DOI:** 10.1089/thy.2019.0719

**Published:** 2020-12-07

**Authors:** Jonathan Louis Gorstein, Jack Bagriansky, Elizabeth N. Pearce, Roland Kupka, Michael B. Zimmermann

**Affiliations:** ^1^ Department of Global Health, University of Washington, Seattle, Washington, USA.; ^2^ Iodine Global Network, Ottawa, Canada.; ^3^ Public Health Nutrition LLC, Atlanta, Georgia, USA.; ^4^ Section of Endocrinology, Diabetes, and Nutrition, Boston University School of Medicine, Boston, Massachusetts, USA.; ^5^ Nutrition Section UNICEF-HQ, New York, New York, USA.; ^6^ Department of Health Sciences and Technology, ETH Zurich, Zurich, Switzerland.

**Keywords:** salt, iodine, goiter, universal salt iodization, iodine deficiency

## Abstract

***Background:*** There has been tremendous progress over the past 25 years to control iodine deficiency disorders (IDDs) through universal salt iodization (USI). In 2019, using the median urinary iodine concentration (MUIC), only 19 countries in the world are classified as iodine deficient; in contrast in 1993, using the total goiter rate (TGR), 113 countries were classified as iodine deficient. However, few analyses have tried to quantify the global health and economic benefits of USI programs, and the shift from TGR to MUIC as the main indicator of IDDs complicates assessment of progress.

***Methods:*** We used a novel approach to estimate the impact of USI on IDDs, applying a regression model derived from observational data on the relationship between the TGR and the MUIC from 24 countries. The model was used to generate hypothetical national TGR values for 2019 based on current MUIC data. TGR in 1993 and modeled TGR in 2019 were then compared for 139 countries, and using consequence modeling, the potential health and economic benefits realized between 1993 and 2019 were estimated.

***Results:*** Based on this approach, the global prevalence of clinical IDDs (as assessed by the TGR) fell from 13.1% to 3.2%, and 720 million cases of clinical IDDs have been prevented by USI (a reduction of 75.9%). USI has significantly reduced the number of newborns affected by IDDs, with 20.5 million cases prevented annually. The resulting improvement in cognitive development and future earnings suggest a potential global economic benefit of nearly $33 billion. However, 4.8 million newborns will be affected by IDDs in 2019, who will experience life-long productivity losses totaling a net present value of $12.5 billion.

***Conclusions:*** The global improvements in iodine status over the past 25 years have resulted in major health and economic benefits, mainly in low- and middle-income countries. Efforts should now focus on sustaining this achievement and expanding USI to reach the continuing large number of infants who remain unprotected from IDDs.

## Introduction

In 1990, the World Summit for Children called for the elimination of iodine deficiency disorders (IDDs) by the year 2000. Four years later, the Joint UNICEF/WHO Committee on Health Policy urged all countries to adopt and implement universal salt iodization (USI) (1). Over the past 25 years, there has been major global progress toward USI and the elimination of IDDs. The proportion of households in low-income countries consuming iodized salt increased from 20% in 1990 to 70% by 2000 and has expanded to 88% in 2019 (2). Today, USI programs are working to consolidate and sustain this achievement and to further expand access to quality iodized salt. The resources needed to support established USI programs are modest compared with those required for implementation in previous decades. However, continued commitment and investment from national politicians, business leaders, and donor organizations remain critical to sustain USI, in a competitive and resource-constrained environment.

Advocacy for USI requires making a compelling case for its benefits, along with a clear description of the remaining IDD burden. However, evidence-based analyses have been difficult, in part, due to a shift in the metrics used to assess program performance and impact. The total goiter rate (TGR) was the recommended indicator of population iodine status for several decades after the first global reviews in 1960 (3). At that time, when endemic goiter due to iodine deficiency was widespread, TGR was a reasonable indicator of moderate-to-severe iodine deficiency (4–6). However, a major limitation of the TGR is that there is a long lag time in the resolution of goiter after iodine intake improves, and in adults, the TGR may represent past, rather than present, IDDs. Thus, its measurement does not accurately capture the changing iodine status of populations and, therefore, a change in the TGR may not reflect the potential contribution of IDDs to decreased intelligence quotient (IQ) and cognitive impairment (7).

Consequently, TGR has given way to the use of urinary iodine concentration (UIC), which is a quantitative and sensitive biochemical indicator that can assess the full range of population iodine status (8). The current indicator recommended to assess national iodine status, the median UIC (MUIC) value, allows for the classification of countries as having optimal iodine nutrition, IDDs, or iodine excess. UIC values are typically based on a single spot urine sample, and these vary substantially between and within individuals, and within individuals during the day. As a result, single spot UIC should not be used to classify an individuals' iodine status, and by extension, should not be used to estimate the proportion of people affected by IDD in a population, or the potential benefits of interventions (9). Defining sufficiency using MUIC does not define the proportion of the population with IDDs. Because of this shift from TGR to MUIC, national USI program baseline and endpoint data may not be consistent, nor are they directly comparable.

Given these restrictions, it has been a challenge to craft simple, compelling, and clearly quantified messages communicating the full health and economic benefits of USI programs—or to describe the remaining burden of IDDs. Consequently, advocacy is often confined to statements about how coverage of iodized salt results in an implied number of newborns “at risk.” Such an approach presumes that an individual covered with iodized salt will not suffer from any degree of deficiency, which is not always the case. Sometimes advocates and IDD program managers have misinterpreted UIC data, incorrectly claiming the proportion of individuals whose UIC values fall below some cutoff point, for example, <100 μg/L, is equivalent to the national prevalence of suboptimal iodine intakes. This approach is inappropriate and leads to artificially inflated estimates of IDDs (10). Similarly, efforts have been made to estimate the number of individuals or newborns unprotected by multiplying the proportion of the population not covered by adequately iodized salt by the total population size or the number of live births, assuming that all newborns without USI protection suffer from IDDs. However, these approaches also likely overestimate the magnitude of the problem and may underestimate the actual impact of salt iodization on the improvement of iodine status.

## Methods

Given the data limitations already outlined, this article proposes a new approach to conceptually describe the full benefits achieved by USI programs between 1993 and 2019 and to concretely estimate the remaining burden of IDDs based on four indicators, namely:

- Reduced prevalence of clinical IDDs between 1993 and 2019.
- Prevented cases of clinical IDDs 1993–2019 (in general population and among newborns).
- Economic benefits of reduced prevalence among newborns suffering intellectual deficits.
- Remaining health and economic burden in populations without access to iodized salt.

The main data sources for this analysis are databases maintained by the Iodine Global Network (IGN) and UNICEF (11). These include data on IDDs before implementation of salt iodization as well as updated information on the iodine status from 195 low- and high-income countries, and the coverage of households with adequately iodized salt. A baseline is generated from an authoritative global review undertaken soon after the World Summit for Children (12). This compiled global, regional, and country TGR estimates, from national surveys undertaken between 1980 and 1993, and from subnational surveys and contextual evidence. To reflect 2019 IDD status, the IGN scorecard provides recent MUIC data for 139 countries gathered in global reviews and include recent population-based surveys (13–15). Based on these sources, this analysis focuses on countries that offer at least one of the data points mentioned, TGR 1993 or MUIC 2019. The analysis grouped countries into the six WHO regions as shown in Table 1. It should be noted that the large number of countries in Africa with estimates in 1993 was based on estimates from the WHO. The larger number of countries in 2019 reflects the increased attention to monitoring of USI programs.

**Table 1.** Countries Included in the Analysis by WHO Region

	No. of countries	
WHO Region	1993	2019
Africa	39	29
Americas	19	22
Eastern Mediterranean	13	19
Europe	39	43
South Asia	9	11
East Asia and Pacific	10	15
Total	129	139

**FIG. 1.**
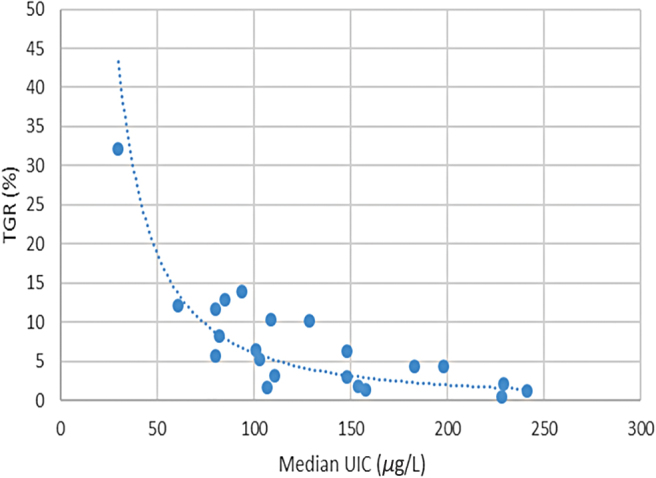
Iodine status data for 24 countries—MUIC among school-age children and total goiter rate. MUIC, median urinary iodine concentration. Color images are available online.

With baseline country-level status derived from TGR and endpoint status based on MUIC, the method required modeling to make these two indicators comparable. It is well established that there is a close correlation between population iodine status based on TGR and MUIC (16). The WHO criteria for the classification of IDDs using UIC data were established based on the observation that an MUIC level of >100 μg/L in a population was usually associated with a <5% TGR, and thus the absence of endemic goiter due to iodine deficiency (16–19). Using information from 24 countries where contemporaneous data exist for both TGR and MUIC, we developed a regression model to estimate TGR levels from MUIC values. This method replicates an approach used by the Instituto de Nutrición de Centro América y Panamá (INCAP), which examined the relationship between TGR and MUIC in 1970 and served as the basis for the 1994 WHO recommendation (17). Data on both UIC and TGR were available from subsets of school-age children included in national iodine status surveys. The data are plotted in Figure 1 and were found to correspond closely to the study undertaken by Ascoli and Arroyave already mentioned, which assessed the association between TGR and UIC from 186 localities in Central America.

Given the nonlinear characteristic of the relationship between the two variables, a power regression model was tested and determined to be the best fit when compared with several other high-order regression models, with the equation

$$y=\alpha x^{\beta },$$

using the natural log of both variables. The resulting model was

TGR = 11,049 × mUIC^−1.63^.

The equation was then used to generate hypothetical national TGR values for 2019 based on current MUIC data for school-age children. This conversion of MUIC to TGR enables a comparison of baseline TGR with the modeled predicted TGR for 2019. Based on the calculated values, TGR 1993 and modeled TGR 2019 were compared for 139 countries, and using the logic model shown in Supplementary Table S1, the potential changes in iodine status and benefits realized between 1993 and 2019 were estimated. Individual parameters for 139 countries were merged into projections for WHO regions. The key outputs from the analysis included:

- Percentage change in TGR prevalence, taken as surrogate indicator for clinical IDDs.
- Cost of Doing Nothing Scenario, derived from applying 1993 TGR to the 2019 population, the number of IDD cases in 2019 in the absence of salt iodization (if TGR 2019 remained at 1993 levels).
- Number of prevented cases, based on the difference between cost of Doing Nothing Scenario and estimated 2019 cases.

The data requirements to drive the consequence modeling were based on the following parameters and assumptions:

- Median earning: National Gross National Income (GNI) per capita from World Bank and estimated wage share (%) taken from a recent analysis at *Institute for Development Policy and Management (IDPM)* at University of Manchester (20).
- Labor force participation rates are taken from World Bank statistics (21).
- Average work life is estimated as the difference between WHO Healthy Life Expectancy and 15 years of age, when work life is projected to commence (22).
- For a child born in 2019, earnings are not projected to begin until 2030 and will stretch decades into the future. Net present value (NPV) is used to define future value in current currency by applying an interest rate of 3% discount rate, commonly used public health analyses (23).

Applying these parameters to the modeled TGR prevalence for 2019 and the cost of Doing Nothing Scenario yielded projections of economic losses attributable to IDDs (Supplementary Table S2). While indicators of IDDs changed from TGR to MUIC, the literature over the past 30 years (including several randomized control trials) continues to consistently show a strong association of both indicators with scores on cognitive tests (24). In 2015, the World Health Organization published a systematic review on the “effect of iodized salt on change in intelligence quotient,” concluding that “children exposed to iodized salt during gestation, infancy and early childhood had higher IQ and reduced risk of low intelligence compared to unexposed children” (25). The median improvement found in the WHO review was 8.18%, suggesting an IQ deficit attributable to IDDs of roughly the same magnitude.

IQ predicts both educational and occupational success even after controlling for income and other indicators of socioeconomic status (26–28). Based on data from eight low-income countries, a recent review derived an average 1.18% earnings deficit for each lost IQ point (29). Given a loose correlation of cognitive test scores of preschool children with IQ scores at 15 years of age, when children are presumed to enter the workforce, a correlation coefficient of 0.64 was applied to these two parameters to suggest that a lifelong economic burden of IDDs, or productivity deficit, may be 6.12% of future earnings (30). This 6.12% coefficient of deficit along with other regional demographic and labor data was applied to estimate the economic consequences for the remaining burden of IDDs for the six World Bank Regions.

## Results

Individual calculations for 129 countries with a total population of 5.1 billion in 1993 and 139 countries with a total population of >7.1 billion in 2019 are summed for each WHO Region. Results given in Table 2 suggest the following key messages for global progress 1993–2019. The global prevalence of clinical IDDs (as assessed by the TGR) fell from 13.1% to 3.2%, with the greatest reduction taking place in the American Region (84.1%), followed by the Eastern Mediterranean Region (83.7%), South Asian Region (78.7%), and the East Asia and Pacific Regions (77.9%). Of note is the slower decline in prevalence observed in Africa and Europe. Even in these regions, however, the absolute number of cases declined by 100 and 70 million, respectively, relative to the Doing Nothing Scenario.

**Table 2.** Summary Global and Regional Iodine Status 1993–2019

	Clinical IDDs 1993			Clinical IDDs 2019			Benefits 1993–2019		
WHO Region	Population	TGR	Affected	Population	TGR	Affected	Doing Nothing Scenario	Prevented cases 2019	Prevalence reduction
	‘000	%	‘000	‘000	%	‘000	Cases ‘000		%
Africa	543,705	15.6	85,029	929,856	4.8	44,716	145,419	100,703	69.3
Americas	691,115	11.0	75,832	970,166	1.7	16,931	106,451	89,520	84.1
Eastern Mediterranean	383,635	24.2	93,004	577,938	3.9	22,808	140,109	117,301	83.7
Europe	782,151	12.8	100,152	907,895	5.1	46,099	116,253	70,154	60.3
South Asia	1,331,968	13.0	172,505	1,878,262	2.8	51,829	243,256	191,427	78.7
East Asia and Pacific	1,403,931	10.5	147,028	1,853,008	2.3	42,978	194,058	151,081	77.9
Total	5,136,505	13.1	673,551	7,117,125	3.2	225,360	945,546	720,186	75.9

IDDs, iodine deficiency disorders; TGR, total goiter rate.

The modeled TGR for 2019 suggests roughly 225 million remaining clinical cases, about one-third of the global burden 2 decades earlier. It should be noted that many of these cases of goiter are likely not due to iodine deficiency, since the prevalence of goiter in most countries does not exceed 5%, and it is assumed that there are other causes, such as normal physiological variation in thyroid size and autoimmune thyroid diseases. Finally, the cost of Doing Nothing Scenario projects a burden of ∼945 million cases, suggesting that 720 million cases of clinical IDDs have been prevented by USI.

**FIG. 2.**
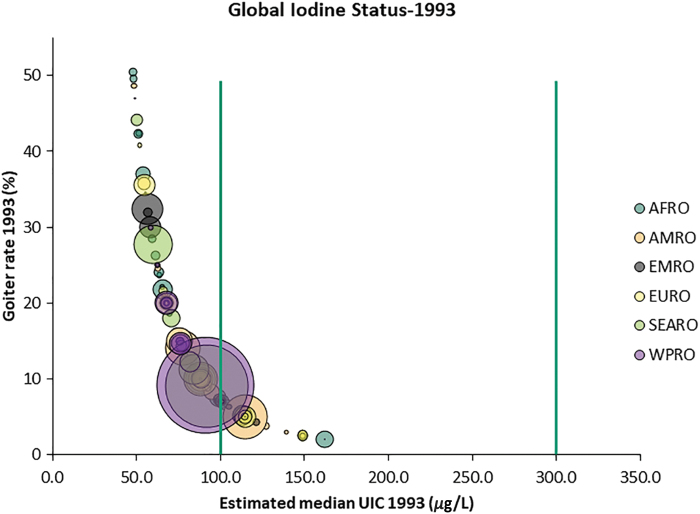
Association between TGR and iodine status in 1993. TGR, total goiter rate. Color images are available online.

**FIG. 3.**
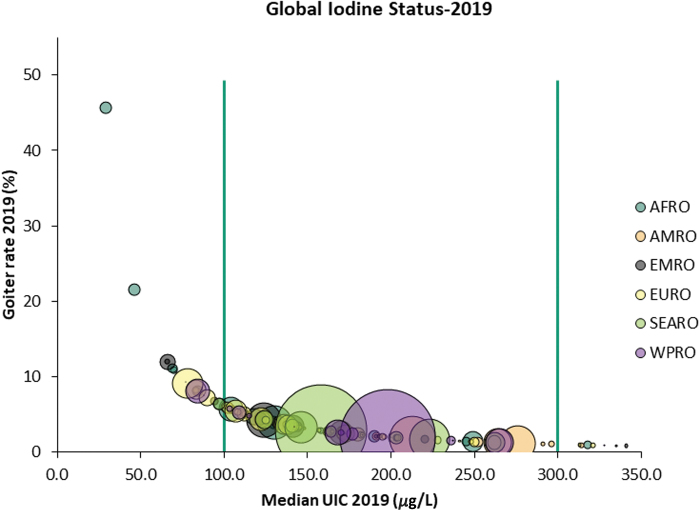
Association between TGR and iodine status in 2019. In Figures 2 and 3, the color of the bubbles corresponds to the WHO region, while the size of the bubbles reflects the size of the population affected. The target for the virtual elimination of clinical IDDs is for a country TGR prevalence to be <5% and an MUIC in the range between 100 and 299 μg/L, representing optimal iodine status at the population level (43). Color images are available online.

Over this period of time, there has been a dramatic decline in the number of countries classified as iodine deficient from 116 countries in 1993 (Fig. 2) to 19 in 2019 (Fig. 3).

Beyond the overall reduction in projected TGR or clinical manifestations of iodine deficiency, the functional benefit of improved iodine status mainly emerges from fewer babies suffering iodine deficiency *in utero* and during infancy—with a reduction in associated cognitive deficits and improved future earnings potential. Of 136 million estimated births in 2019 among the 6 WHO regions, the calculations shown in Table 3 suggest 4.8 million IDD cases might be expected annually at current TGR. In contrast, the cost of Doing Nothing Scenario (applying 1993 TGR to 2019 births) indicates ∼25.3 million cases, a difference of almost 20.5 million cases prevented annually.

**Table 3.** Estimations for Newborns with Iodine Deficiency Disorders: Total Goiter Rate 1993 and Modeled Total Goiter Rate 2019

	Births	TGR	Newborns w/IDDs
	‘000	%	‘000
IDD cases: 2019 modeled TGR prevalence			
Africa	33,681	4.8	1740
Americas	15,074	1.7	272
Eastern Mediterranean	15,406	3.9	636
Europe	11,119	5.1	559
South Asia	36,518	2.8	1010
East Asia and Pacific	24,350	3.1	609
Total	136,149	3.2	4825
IDD cases: cost of Doing Nothing Scenario (based on 1993 data)			
Africa	38,898	15.6	6341
Americas	20,181	11.0	2511
Eastern Mediterranean	17,773	24.2	4307
Europe	10,445	12.8	1748
South Asia	53,128	13.0	6589
East Asia and Pacific	33,538	10.5	3793
Total	173,963	13.1	25,288

The economic benefits attributable to USI are described in Table 4. Key messages emerging from this economic projection include the fact that if there had been no improvement in iodine status in the period between 1993 and 2019, >25 million newborns would be afflicted with IDDs, leading to annual global losses of $45.2 billion (NPV). The improved iodine status, emerging mainly from the achievement of salt iodization in these 159 countries, represents an economic benefit of nearly $32.2 billion annually. Finally, there are 4.8 million newborns still suffering IDDs in 2019 who will experience life-long productivity losses totaling NPV $12.5 billion in 2019.

**Table 4.** Net Present Value of Losses Attributable to Iodine Deficiency Disordersat Two Rates: Modeled Total Goiter Rate for 2019 and Total Goiter Rate 1993

	Newborns w/IDDs	Labor	Annual	NPV losses
	‘000	Participation (%)	Income (US$)	@ 3% (,000)
Current Iodine Status @ modeled TGR 2019				
Africa	1740	71.8	1146	838,880
Americas	272	65.1	5722	3,246,795
Eastern Mediterranean	636	55.1	8100	794,755
Europe	559	59.3	18,596	4,683,995
South Asia	1010	69.4	1479	433,285
East Asia and Pacific	609	64.0	6372	2,485,492
Total	4825			12,483,202
Cost of Doing Nothing @ 1993 TGR				
Africa	6341	70.2	1146	1,326,288
Americas	2511	62.4	5722	14,736,522
Eastern Mediterranean	4307	52.8	8435	3,463,865
Europe	1748	59.3	18,596	8,247,588
South Asia	6589	68.3	1479	3,092,915
East Asia and Pacific	3793	67.4	6372	14,353,332
Total	25,288			45,220,509

NPV, net present value.

## Discussion

This new analysis estimates the impact over the past 25 years toward the global elimination of severe iodine deficiency, primarily through salt iodization programs that have increased the supply of adequately iodized salt throughout the world. This includes both the salt that is used for discretionary purposes at the household level and the salt used in the manufacture of processed foods and condiments. However, there are some limitations of this study, as discussed hereunder.

The projections are based on modeling of the association between the TGR and the mUIC, two population metrics related to iodine status, and assumes that all goiters are the direct consequence of iodine deficiency, which is likely not the case: a small number will be due to other causes, including autoimmune thyroid disease (31–33). The methodology may underestimate the prevalence of IDDs and the scale of the associated intellectual disability, which, in turn, may compromise projections of the economic deficit resulting from suboptimal iodine intake. Goiter does not capture the full range of IDD disability, in particular the wide occurrence of mild IDDs that has been associated with IQ deficits (34–36). TGR, assessed by physical palpation, measures only moderate-to-severe forms of IDDs (35,37). In contrast, the UIC is considered a much more sensitive quantitative indicator of current iodine intake. Since goiter is generally associated with more serious degrees of deficiency, the TGR derived in this algorithm likely reflects more pronounced clinical cases of IDDs. Therefore, the analysis is likely a conservative assessment of USI benefits.

Although the MUIC data used in this analysis are the most recent estimates of iodine status from nationally representative surveys, some of the data points are >10 years old. Consequently, while they provide the best gauge of current status, the situation may have changed since the time of the surveys. Furthermore, our analysis is based on a discordant number of countries: 129 in 1993 and 139 from 2019. As such, the estimates on the impact of USI on goiter and economic benefits are likely to be underestimated, since the absolute magnitude of the baseline problems reflects a subgroup of countries for which data are available in 2019.

There is ongoing research to develop a feasible and robust approach to describe the prevalence of IDDs and suboptimal iodine intake based on replicate UIC spots samples and adjustment for creatinine, age, sex, and/or body weight from individuals (38). As these methods and tools are refined, the approach outlined here will enable an estimation of the number and proportion of individuals with clinical IDDs based on current population surveys and compare these with earlier global figures.

The current analysis suggests that, as a result of USI programs, significant improvements in iodine status have reduced the burden of clinical iodine deficiency and led to tangible social and economic benefits. This study confirms earlier analyses of the economic benefits of salt iodization by the WHO (39,40) and the Copenhagen Consensus (41), which have estimated a benefit–cost ratio of the order of 30:1, although others have suggested that the benefit–cost ratio may be as high as 70:1 (42). This analysis extends those earlier projections to consider the impact of salt iodization on the goiter prevalence and parameters of economic development at the national, regional, and global levels. The projected loss of $45 billion in all countries due to iodine deficiency in the absence of salt iodization is comparable with the figure estimated by Horton of $35.7 billion (45) before salt iodization that only included low-income countries.

Our analysis suggests that the global increase in population coverage with iodized salt from 1993 to 2019 has produced significant economic returns, along with major improvements in population health. The successful implementation of salt iodization programs has required the support of multiple stakeholders and partners. As such, it is imperative to maintain current efforts and sustain achievements, but also to determine where more intensive efforts may be warranted to ensure that all countries have viable and sustained USI programs that reach all segments of the population. This requires continued and additional political commitment and national investment, improved regulatory monitoring, and harmonization with the broader nutrition agenda.

## Supplementary Material

Supplemental data
