# Supplementary material for: Estimating the Health and Economic Benefits of Universal Salt Iodization Programs to Correct Iodine Deficiency Disorders
Source: Thyroid. 2020 Dec 7;30(12):1802–9. doi: 10.1089/thy.2019.0719 (PMC7757618; doi:10.1089/thy.2019.0719)
Supplement: Supplemental data [file Supp_Tables1-2.pdf]

Supplementary Data

SUPPLEMENTARY TABLE S1. LOGIC MODEL TO DEVELOP BENEFITS

Clinical IDD 1993			*Clinical IDD 2019*			*Benefits 1993–2019*		
a	*b*	*c*	*d*	*e*	*f*	*g*	*h*	*i*
Population	TGR %	Baseline cases	Population	Derived TGR %	Endline cases	Improved prevalence	Cases do nothing scenario	Prevented or saved cases
UN population	IGN database	a×b	UN population	IGN database	d×e	(b − e)/b	b×d	h – f

IDD, iodine deficiency disorders; IGN, Iodine Global Network; TGR, total goiter rate.

SUPPLEMENTARY TABLE S2. LOGIC MODEL AND PARAMETERS TO PROJECT BASELINE ECONOMIC LOSSES

Coefficient of deficit		*Number affected*		*Labor force participation*		*Median earning*		*Average work-life*		*Apply NPV*		*Losses to economy*
% Earnings deficit: 6.12%	×	TGR% × # births	×	%	×	$/year: GNI ×wage share	×	15–65 years: 50 years		NPV @3%	=	NPV $/year

NPV, net present value.
